# Joint modelling rationale for chained equations

**DOI:** 10.1186/1471-2288-14-28

**Published:** 2014-02-21

**Authors:** Rachael A Hughes, Ian R White, Shaun R Seaman, James R Carpenter, Kate Tilling, Jonathan AC Sterne

**Affiliations:** 1 School of Social and Community Medicine, University of Bristol, Bristol, UK; 2 MRC Biostatistics Unit, Institute of Public Health, Cambridge, UK; 3 London School of Hygiene and Tropical Medicine, London, UK; 4 MRC Clinical Trials Unit, London, UK

**Keywords:** Chained equations imputation, Gibbs sampling, Joint modelling imputation, Multiple imputation, Multivariate missing data

## Abstract

**Background:**

Chained equations imputation is widely used in medical research. It uses a set of conditional models, so is more flexible than joint modelling imputation for the imputation of different types of variables (e.g. binary, ordinal or unordered categorical). However, chained equations imputation does not correspond to drawing from a joint distribution when the conditional models are incompatible. Concurrently with our work, other authors have shown the equivalence of the two imputation methods in finite samples.

**Methods:**

Taking a different approach, we prove, in finite samples, sufficient conditions for chained equations and joint modelling to yield imputations from the same predictive distribution. Further, we apply this proof in four specific cases and conduct a simulation study which explores the consequences when the conditional models are compatible but the conditions otherwise are not satisfied.

**Results:**

We provide an additional “non-informative margins” condition which, together with compatibility, is sufficient. We show that the non-informative margins condition is not satisfied, despite compatible conditional models, in a situation as simple as two continuous variables and one binary variable. Our simulation study demonstrates that as a consequence of this violation order effects can occur; that is, systematic differences depending upon the ordering of the variables in the chained equations algorithm. However, the order effects appear to be small, especially when associations between variables are weak.

**Conclusions:**

Since chained equations is typically used in medical research for datasets with different types of variables, researchers must be aware that order effects are likely to be ubiquitous, but our results suggest they may be small enough to be negligible.

## Background

Multiple imputation [1] has become a popular approach for the analysis of incomplete data, with several mainstream statistical packages now incorporating multiple imputation tools. It involves making several draws of the missing data from their posterior predictive distribution given the observed data and an imputation model. For multivariate, non-monotone missing data there are two main approaches for constructing an imputation model: joint modelling and chained equations. Joint modelling imputation requires the specification of a parametric joint model for the complete data: current implementations impute under the multivariate normal model, the log linear model and the general location model [2]. However for datasets containing different types of variables the current classes of joint models [3-5] may not be appropriate for the joint distribution of the data. The alternative method, chained equations imputation [4,6], is more flexible as it specifies a separate imputation model, typically a univariate regression model, for each incomplete variable and updates the missing data for each variable in turn.

Chained equations imputation has been proposed under several different names including: fully conditional specification, stochastic relaxation, variable-by-variable imputation, regression switching, sequential regressions, ordered pseudo-Gibbs sampler, partially incompatible MCMC and iterated univariate imputation [3]. In addition to handling variables of varying types, the chained equations approach has other flexible features such as incorporating restrictions, logistical and consistency bounds (for example, to handle imputation of gender specific variables or impute only questions that were not intentionally skipped in a questionnaire [4]). van Buuren and Groothuis-Oudshoorn [7] discuss the wide range of medical fields that have used chained equations imputation (e.g. addiction [8], epidemiology [9], infectious diseases [10], genetics [11], cancer [12], obesity and physical activity [13]), and a brief review of available software that have implemented chained equations imputation is given by [14]. Given the popularity of chained equations, among users of varying degrees of expertise, there is now guidance in its practical use (e.g. [14] and [15]).

Despite its widespread use, a known theoretical weakness of the chained equations method is that the implicit joint distribution underlying the separate models may not always exist: that is, the conditional models may be incompatible [4,5,16-18]. In such situations, the results after chained equations imputation may systematically differ according to the order in which the missing variables are updated in the chained equations algorithm. We shall refer to this phenomenon as an “order effect”.

Previous authors [3-5] have stated that chained equations imputation under a set of normal linear regression models, with all other variables as covariates and no interactions, is equivalent to a Gibbs sampler that draws from a multivariate normal distribution. van Buuren [3] also states for a dataset of three partially observed binary variables that chained equations under a set of logistic regression models, with all other variables included as main effects only, is equivalent to a joint modelling imputation under a log linear model with the three-way factor term set to zero. However, none of these papers provides a proof beyond stating that the set of conditional models is compatible [16] and are all derived from the specified joint distribution.

Independently and concurrently with our work, Liu et al. [19] have given sufficient conditions (which include compatibility of the conditionals) under which, as the sample size tends to infinity, the stationary distribution of the Markov chain generated by the chained equations algorithm (assuming that this stationary distribution exists and that the chain converges to it) converges to the posterior predictive distribution of the missing data implied by a joint Bayesian model. That is, under these sufficient conditions, the total variation of the distance between the chained equations stationary distribution and the posterior predictive distribution tends to zero as the sample size tends to infinity. As a corollary, Liu *et al* show the equivalence of the two imputation methods in finite samples under a condition we have independently identified and named the “non-informative margins” condition.

Our work is complementary to that of Liu et al. Firstly, we have taken a different approach to prove the equivalence of the two imputation methods in finite samples. Additionally, in specific examples, we prove whether the non-informative margins condition is satisfied or not, and in a simulation study we demonstrate the consequences when the conditional models are compatible but do not satisfy the non-informative margins condition.

In this paper, we provide a “non-informative margins” condition that, together with compatibility of the conditionals (and assuming that the Markov chain generated by the chained equations converges to a stationary distribution), guarantees that the imputed values obtained using chained equations (at convergence) are drawn from the posterior predictive distribution of the missing data implied by a Bayesian joint model. We give examples of chained equations algorithms that satisfy the non-informative margins condition when the joint model is the multivariate normal model and the saturated multinomial model, and examples where this condition is not satisfied when the joint model is an unsaturated multinomial model and the general location model. A simulation study considers a simple chained equations algorithm in which the conditional models are compatible but do not satisfy the non-informative margins condition, and shows that it is not equivalent to any joint model procedure.

## Methods

### Notation

Suppose *K* random variables $X={\left(X_{1},\ldots ,X_{K}\right)}^{⊺}$ are intended to be observed on *N* subjects. We use subscripts *i* and *j* to index subjects and variables respectively (*i* = 1,…,*N*; *j* = 1,…,*K*). Let *x* = (*x*_*ij*_) denote an (*N* × *K*) matrix, whose *i*,*j* element is *x*_*ij*_. Column *j* of matrix *x* is denoted by $x_{j}={\left(x_{1j},\ldots ,x_{\text{Nj}}\right)}^{⊺}$. It is assumed that the rows of matrix *x* are independent and identically distributed draws from a probability distribution with probability distribution function *p* (*X* ∣ *θ*), where *θ* is an unknown parameter.

In practice some subjects have missing observations on up to *K* - 1 variables and we write *x*_*j *_= (*xjobs*,*xjmis*) for any *j*, *x* = (*x*^*obs*^,*x*^*mis*^) and *p* (*x* ∣ *θ*) = *p*(*x*^*obs*^,*x*^*mis*^ ∣ *θ*), with superscript *obs* and *mis* denoting the observed and missing data respectively. In keeping with the assumptions of joint modelling imputation and chained equations imputation, the missing data mechanism is assumed to be ignorable for Bayesian inference [20] p. 120, so that inferences about *θ* can be based on the marginal observed data posterior *p* (*θ* ∣ *x*^*obs*^).

### Joint modelling imputation

Joint modelling imputation requires the specification of a parametric joint model *p*(*x*^*obs*^,*x*^*mis* ^∣ *θ*) for the complete data and a prior distribution *p*(*θ*) for parameter *θ*. Imputations are independent draws from the posterior predictive distribution of the missing data given the observed data *p*(*x*^*mis* ^∣ *x*^*obs*^) [2] p. 105, which under the ignorability assumption is

$$p\left(x^{\text{mis}}|x^{\text{obs}}\right)=\int p\left(x^{\text{mis}}|x^{\text{obs}},\theta \right)p\left(\theta |x^{\text{obs}}\right)\mathrm{d\theta .}$$

Therefore, to draw from this posterior predictive distribution, first draw *θ*^∗^ ∼ *p* (*θ* ∣ *x*^*obs*^) followed by *x*^*mis*∗^ ∼ *p* (*x*^*mis*^ ∣ *x*^*obs*^,*θ*^∗^) [2] p. 105. When it is difficult to draw from the observed data posterior *p*(*θ*∣*x*^*obs*^), Markov chain Monte Carlo methods can be used. For example, the data augmentation algorithm of Tanner and Wong [21] draws missing values from the posterior predictive distribution *x*^*mis*∗^ ∼ *p* (*x*^*mis* ^∣ *x*^*obs*^,*θ*^∗^) and then draws *θ* from the complete data posterior *θ*^∗^ ∼ *p* (*θ* ∣ *x*^*obs*^,*x*^*mis*∗^), where ∗ denotes the last drawn values of *θ* or *x*^*mis*^. Upon convergence this produces a draw from the joint posterior distribution *p*(*θ*,*x*^*mis*^ ∣ *x*^*obs*^).

### Chained equations imputation

For every incomplete variable the chained equations algorithm requires an imputation model, typically a univariate regression model, and an accompanying prior distribution for the model’s parameter. Let *X*_-*j*_ = (*X*_1_,…,*X*_*j*-1_,*X*_*j*+1_,…,*X*_*K*_)^T^ denote the vector of random variables excluding variable *X*_*j*_ and $x_{-j}=(x_{-j}^{\text{obs}},x_{-j}^{\text{mis}})$ the submatrix of *x* corresponding to variables *X*_-*j*_. We write *p* (*x*_*j*_ ∣ *x*_-*j*_,*ψ*_*j*_) for the probability distribution function of the imputation model for variable *X*_*j *_and *p* (*ψ*_*j*_) for the prior distribution of the unknown parameter *ψ*_*j*_.

Chained equations draws the imputations using an iterative algorithm, typically with 10 to 20 iterations [15]. To start off, the missing values of each incomplete variable are replaced by its mean or a random sample of its observed values. Suppose, without loss of generality, that variables *X*_1_,…,*X*_*R*_ (*R* ≤ *K*) are incomplete and variables *X*_*R*+1_,…,*X*_*K*_ are fully observed. Given the imputations from the last iteration $\left(x_{1}^{\left(t-1\right)},\ldots ,x_{R}^{\left(t-1\right)}\right)$, iteration *t* of the chained equations algorithm consists of the following draws [18]

$$\;\begin{array}{l} \psi_{1}^{\left(t\right)}∼p(\psi_{1})p\;\left(x_{1}^{\text{obs}}|x_{2}^{\left(t-1\right)},x_{3}^{\left(t-1\right)},\ldots ,x_{R}^{\left(t-1\right)},x_{R+1},\ldots ,x_{K},\psi_{1}\right)\\ x_{1}^{\text{mis}(t)}∼p\left(x_{1}^{\text{mis}}|x_{2}^{\left(t-1\right)},x_{3}^{\left(t-1\right)},\ldots ,x_{R}^{\left(t-1\right)},x_{R+1},\ldots ,x_{K},\psi_{1}^{\left(t\right)}\right)\\ \psi_{2}^{\left(t\right)}∼p(\psi_{2})p\left(x_{2}^{\text{obs}}|x_{1}^{\left(t\right)},x_{3}^{\left(t-1\right)},\ldots ,x_{R}^{\left(t-1\right)},x_{R+1},\ldots ,x_{K},\psi_{2}\right)\\ x_{2}^{\text{mis}(t)}∼p\left(x_{2}^{\text{mis}}|x_{1}^{\left(t\right)},x_{3}^{\left(t-1\right)},\ldots ,x_{R}^{\left(t-1\right)},x_{R+1},\ldots ,x_{K},\psi_{2}^{\left(t\right)}\right)\\ \vdots \\ \psi_{R}^{\left(t\right)}∼p(\psi_{R})p\left(x_{R}^{\text{obs}}|x_{1}^{\left(t\right)},x_{2}^{\left(t\right)},\ldots ,x_{R-1}^{\left(t\right)},x_{R+1},\ldots ,x_{K},\psi_{R}\right)\\ x_{R}^{\text{mis}(t)}∼p\left(x_{R}^{\text{mis}}|x_{1}^{\left(t\right)},x_{2}^{\left(t\right)},\ldots ,x_{R-1}^{\left(t\right)},x_{R+1},\ldots ,x_{K},\psi_{R}^{\left(t\right)}\right). \end{array}$$

During each iteration the following two steps are applied to each incomplete variable *X*_*j*_ in turn: $\psi_{j}^{*}$ is drawn from the posterior distribution proportional to $p(\psi_{j})p(x_{j}^{\text{obs}}|x_{-j}^{*},\psi_{j})$ and missing values $x_{j}^{\text{mis}*}$ are drawn from the predictive posterior $p(x_{j}^{\text{mis}}|x_{-j}^{*},\psi_{j}^{*})$. The imputations from the last iteration form the imputed dataset. The whole iterative algorithm is repeated to obtain further imputed datasets.

### Equivalence of joint modelling and chained equations imputation

We investigated, in finite samples, sufficient conditions under which a chained equations algorithm with compatible conditional models imputes missing data from the predictive distribution of the missing data implied by the joint model and its accompanying prior. We provide examples of chained equations algorithms (with compatible conditional models) where our identified condition is satisfied and examples where it is not satisfied.

### Simulation study

We conducted a simulation study to explore the consequences for chained equations imputation when the conditional models were compatible with the same joint model but the non-informative margins condition of Proposition 1 was not satisfied. In particular, we looked for evidence of “order effects”, where the distribution from which the final imputed values of the variables were drawn differed according to the order in which the variables were updated in the chained equations sampler. If the chained equations algorithm imputes all variables from the predictive distribution of the missing data implied by a specific joint model, then order effects cannot occur [22]. Thus, the existence of order effects implies that the chained equations algorithm is not equivalent to imputing from any joint model.

The simulation study was based on a general location model, discussed in the Theoretical results section below, with one incomplete binary variable *Y* and two continuous variables *W*_1_ and *W*_2_, where *W*_1_ was also incompletely observed. We compared joint modelling imputation under the general location model, considered as a gold standard, with the chained equations algorithm that imputes the binary variable *Y* under a logistic regression model and the continuous variable *W*_1_ under a normal linear regression model.

We generated 500 datasets, each with a sample size of 100. For each dataset, the rows were independent, identically distributed realizations of the general location model *Y* ∼ Bernoulli (3/10), *W*_1_ ∣ *Y* ∼ *N* (10 + *β* *Y*,9) and *W*_2_ ∣ *W*_1_,*Y* ∼ *N* (9 + 8/9 + 1/9*W*_1_ + *βY*,8 + 8/9). The data model was a simplified version of data that can occur in the medical literature [23]. The simulation study was repeated when *β*, the regression coefficient for covariate *Y*, was set to 1 and 3. The analysis of interest was the normal linear regression of *W*_2_ on *W*_1_ and *Y*. To ensure that any observed order effects could only be due to the failure of the non-informative margins condition we considered the simplest setting, that of data missing completely at random [20] p. 16, and set the values of *Y* and *W*_1_ to be missing for the first 50 individuals in the dataset. Below we describe the joint modelling imputation procedure and the chained equations algorithm that were separately applied to the same 500 datasets.

We used the data augmentation algorithm (as described under the heading “Joint modelling imputation”) to perform joint modelling imputation under the general location model and the joint prior given in the general location example (see example 4 of the Results), setting hyperparameters *τ* = *ν* = 1/2 and *κ* = 3/2. The number of imputed datasets generated, the burn-in period and the number of iterations between imputed datasets was 100. The analysis model was applied to each dataset separately and the mean of the multiple estimates of *β*, the coefficient for *Y*, was calculated.

In the (standard) chained equations algorithm, a logistic regression model for *Y* given *W*_1_ and *W*_2_ was first fitted to those rows of the dataset in which *Y* was observed. Let ${\hat{\psi }}_{Y}$ denote the maximum likelihood estimate of the parameters of this model and $\hat{V}$ denote its associated estimated variance-covariance matrix. A draw $\psi_{Y}^{*}$ was then made from the multivariate normal approximation $N({\hat{\psi }}_{Y},\hat{V})$ and used to impute the missing *Y* values. The continuous variable *W*_1_ was imputed using the linear regression model *W*_1_ ∣ *Y*,*W*_2_ ∼ *N* (*λ* + *ξY* + *ϕW*_2_,*ω*) and prior distribution *p* (*λ*,*ξ*,*ϕ*,*ω*) ∝ *ω*^-3/2^.

To start off the chained equations algorithm the missing values of *Y* and *W*_1_ were replaced with a random sample of their observed values. We augmented the chained equations algorithm such that, within each iteration we fitted the analysis model immediately after updating the binary variable *Y* and also immediately after updating the continuous variable *W*_1_. The simulation study focused on systematic differences between the two resulting estimates of *β*. Given the imputations from the last iteration $\left(y^{\left(t-1\right)},w_{1}^{\left(t-1\right)}\right)$, iteration *t* of the augmented chained equations algorithm consisted of the following steps: 

1. Generate *y*^(*t*)^ by imputing values for the missing binary observations, conditioning on $w_{1}^{\left(t-1\right)}$ and *w*_2_.

2. Linearly regress *w*_2_ on $w_{1}^{\left(t-1\right)}$ and *y*^(*t*)^ and store the estimate for the coefficient of *Y*, denoted by ${\hat{\beta }}^{b}$.

3. Generate $w_{1}^{\left(t\right)}$ by imputing values for the missing continuous observations, conditioning on *y*^(*t*)^ and *w*_2_.

4. Linearly regress *w*_2_ on $w_{1}^{\left(t\right)}$ and *y*^(*t*)^ and store the estimate for the coefficient of *Y*, denoted by ${\hat{\beta }}^{c}$.

The chained equations algorithm was implemented with 10010 iterations. The first 10 iterations were regarded as burn-in and the estimates from these iterations discarded. The remaining 10000 estimates of ${\hat{\beta }}^{b}$ were averaged, and likewise for ${\hat{\beta }}^{c}$. We denote these means as ${\bar{\beta }}^{b}$ and ${\bar{\beta }}^{c}$ and their difference by ${\bar{\beta }}^{b}-{\bar{\beta }}^{c}$. The quantity ${\bar{\beta }}^{b}-{\bar{\beta }}^{c}$ can be interpreted as an estimate of the order effect for imputation in one dataset. We estimated the (Monte Carlo) standard error of ${\bar{\beta }}^{b}-{\bar{\beta }}^{c}$ using the batch-means method, a method for computing standard errors for correlated output [24] p. 124, and calculated a 95% confidence interval from this.

Linear discriminant analysis is an alternative way to estimate a logistic regression [25,26]. A modified chained equations algorithm using linear discriminant analysis on all individuals with observed *Y* has been proposed as an alternative way to impute the binary variable *Y*[27]. Because the linear discriminant likelihood is the joint distribution of *Y*, *W*_1_ and *W*_2_, this model has the advantage of recovering some information about *ψ*_*Y*_ in the *W* margin. We repeated the simulation study using this modified chained equations algorithm.

We assessed the sensitivity of our results by repeating the simulation study using different specifications. For joint modelling imputation we increased the number of imputed datasets generated, the burn-in period and the number of iterations between imputed datasets to 250. For the standard and modified chained equations procedures we (1) increased the burn-in period of the chained equations sampler to 1000 iterations and (2) sampled every 50^*th*^ iteration thereby reducing serial correlation (with a burn-in period of 10 iterations). To check that our results were not dependent upon our choice of prior distributions we repeated the simulation study with improper imputation procedures; that is, using maximum likelihood estimates of *ψ*_*j*_ instead of Bayesian draws *ψ*_*j*_ from its posterior distribution $p(\psi_{j})p(x_{j}^{\text{obs}}|x_{-j}^{*},\psi_{j})$. Lastly, we also repeated the simulation study with a sample size of 1000 observations.

## Results

### Theoretical results

#### ***Equivalence of joint modelling and chained equations imputation***

In this section, we give our key result Proposition 1, which shows that, in finite samples, compatibility of the conditionals and our proposed non-informative margins condition are sufficient for chained equations and joint modelling to yield imputations from the same predictive distribution.

Consider a joint model *p* (*x* ∣ *θ*) and prior *p* (*θ*). From here onwards we shall use *p* (.∣.) to refer to any probability distribution derived from this joint model. In particular, for each *j* = 1,…,*R*, *p* (*x*_*j *_∣ *x*_-*j*_,*θ*) is the conditional distribution of *x*_*j *_given *x*_-*j *_and *θ* implied by the joint model, and *p*(*x*_-*j *_∣ *θ*) is the conditional distribution of *x*_-*j *_given *θ*. The distribution of *x* given *θ* factorizes as

$$p(x|\theta )=p(x_{j}|x_{-j},\theta )p(x_{-j}|\theta ).$$

Let *ψ*_*j *_and ${\tilde{\psi }}_{j}$ be functions of *θ* such that *p* (*x*_*j *_∣ *x*_-*j*_,*θ*) = *p*(*x*_*j *_∣ *x*_-*j*_,*ψ*_*j*_) and $p(x_{-j}|\theta )=p(x_{-j}|{\tilde{\psi }}_{j})$. That is, the distribution of *x*_*j *_given *x*_-*j *_and the distribution of *x*_-*j *_depend on *θ* only through the functions *ψ*_*j *_and ${\tilde{\psi }}_{j}$, respectively, of *θ*. Let $p(\psi_{j},{\tilde{\psi }}_{j})$ denote the joint prior for *ψ*_*j *_and ${\tilde{\psi }}_{j}$ implied by *p* (*θ*), and let *p* (*ψ*_*j*_) and $p({\tilde{\psi }}_{j})$ denote the corresponding marginal priors.

The chained equations algorithm applies the following two steps for each *x*_*j *_in turn: ***Step CE1 *** Draw $\psi_{j}^{*}$ from the distribution proportional to $p(\psi_{j})p(x_{j}^{\text{obs}}|x_{-j}^{*},\psi_{j})$. **Step CE2** Draw $x_{j}^{\text{mis}*}$ from $p(x_{j}^{\text{mis}}|x_{-j}^{*},\psi_{j}^{*})$.

The choice of the parameterizations *ψ*_*j *_and ${\tilde{\psi }}_{j}$ does not affect the output of step CE2, but a parsimonious choice will help to make the condition of Proposition 1 hold.

##### 

**Proposition 1.** Upon convergence, the chained equations algorithm defined by CE1 and CE2 and the joint model *p* (*x* ∣ *θ*) and prior *p* (*θ*) draw from the same predictive distribution of *x*^*mis *^if, for each incomplete variable *x*_*j*_, $p(\psi_{j},{\tilde{\psi }}_{j})=p(\psi_{j})p({\tilde{\psi }}_{j})$, i.e. if the joint prior distribution for *ψ*_*j *_and ${\tilde{\psi }}_{j}$ factorizes into independent priors for *ψ*_*j *_and ${\tilde{\psi }}_{j}$.

##### 

**Proof.** Using the condition of Proposition 1,

$$\;\begin{array}{ll} p\left(\;\psi_{j},{\tilde{\psi }}_{j}|x_{j}^{\text{obs}},x_{-j}^{*}\;\right) & \propto p\left(\psi_{j},{\tilde{\psi }}_{j}\right)p\left(x_{j}^{\text{obs}},x_{-j}^{*}|\psi_{j},{\tilde{\psi }}_{j}\right)\\ & =p(\psi_{j})p({\tilde{\psi }}_{j})p\left(x_{j}^{\text{obs}}|x_{-j}^{*},\psi_{j}\right)p\left(x_{-j}^{*}|{\tilde{\psi }}_{j}\right) \end{array}$$

Now, integrating out ${\tilde{\psi }}_{j}$,

$$p\left(\psi_{j}|x_{j}^{\text{obs}},x_{-j}^{*}\right)\propto p(\psi_{j})p\left(x_{j}^{\text{obs}}|x_{-j}^{*},\psi_{j}\right)$$

Therefore, step CE1 yields a draw from $p(\psi_{j}|x_{j}^{\text{obs}},x_{-j}^{*})$.

Next, $x_{j}^{\text{mis}}$ and $x_{j}^{\text{obs}}$ are conditionally independent given $x_{-j}^{*}$ and $\psi_{j}^{*}$ so $p(x_{j}^{\text{mis}}|x_{-j}^{*},\psi_{j}^{*})=p(x_{j}^{\text{mis}}|x_{j}^{\text{obs}},x_{-j}^{*},\psi_{j}^{*})$ and step CE2 yields a draw from $p(x_{j}^{\text{mis}}|x_{j}^{\text{obs}},x_{-j}^{*},\psi_{j}^{*})$.

Hence steps CE1 and CE2 together yield a draw from $p(\psi_{j},x_{j}^{\text{mis}}|x_{j}^{\text{obs}},x_{-j}^{*})$, and in particular they draw $x_{j}^{\text{mis}*}$ from $p(x_{j}^{\text{mis}}|x_{j}^{\text{obs}},x_{-j}^{*})$. The latter is a full-conditional distribution corresponding to the joint density *p* (*x*) implied by the joint model. Once $x_{j}^{\text{mis}*}$ has been sampled, $\psi_{j}^{*}$, the sampled value of *ψ*_*j*_, is not used again in the chained equations algorithm. So, the application of steps CE1 and CE2 to each *j* in turn and then iterating is a Gibbs sampler which, at convergence, yields a draw from *p*(*x*^*mis *^∣ *x*^*obs*^), the predictive distribution implied by the joint model and its accompanying prior.

**Comment 1.** The condition of Proposition 1 does not hold if the conditional and marginal parameters *ψ*_*j*_ and ${\tilde{\psi }}_{j}$ are not distinct (i.e. if their joint parameter space is not the product of their separate parameter spaces), and in particular if the combined dimension of *ψ*_*j *_and ${\tilde{\psi }}_{j}$ is greater than that of *θ*. Distinctness of parameter spaces is a property of the model *p* (*x* ∣ *θ*) and not of the prior *p* (*θ*). It will be used in the examples of the unsaturated multinomial distribution and the general location model to identify joint models where the condition of Proposition 1 does not hold. **Comment 2.** Heuristically, the condition of Proposition 1 says that there is no information about *ψ*_*j *_ in the marginal distribution of *x*_-*j*_, so we call it the “non-informative margins” condition. When such information does exist, it is used by the joint modelling sampler but not by the chained equations algorithm, so they may draw from different distributions. Our simulation study will show that this occurs in an example by demonstrating order effects. **Comment 3.** As the non-informative margins condition has only been shown to be sufficient, then potentially if this condition is not satisfied the chained equations algorithm defined by CE1 and CE2 and the joint model *p* (*x*∣*θ*) and prior *p* (*θ*) could still draw from the same predictive distribution of *x*^*mis*^. **Comment 4.** This proof holds for improper prior distributions provided the posterior distributions are proper. **Comment 5.** When the non-informative margins condition holds true the chained equations algorithm is a Gibbs sampler, and so order effects cannot occur [22].

#### ***Example 1: Multivariate normal***

Consider the multivariate normal joint model *X* ∼ *N* (*μ*,*Σ*) with parameter *θ* = (*μ*,*Σ*) and prior distribution *p* (*θ*) ∝ |*Σ* | ^-*κ*^(κ∈ℚ). We show that the corresponding chained equations algorithm, which imputes under a set of normal linear regression models, satisfies the non-informative margins condition of Proposition 1 (and hence draws from the same joint model as joint modelling imputation).

For each *j* we partition the mean vector *μ* as ${\left(\mu_{j},{\tilde{\mu }}_{j}\right)}^{\text{T}}$ and the covariance matrix *Σ* as

$$\left[\begin{array}{cc} \sigma_{j} & \varsigma_{j}^{\text{T}}\\ \varsigma_{j} & {\tilde{\Sigma }}_{j} \end{array}\right],$$

such that *X*_*j *_∼ *N* (*μ*_*j*_,*σ*_*j*_) and $X_{-j}∼N({\tilde{\mu }}_{j},{\tilde{\Sigma }}_{j})$. The conditional distribution of *X*_*j *_given *X*_-*j *_is the normal linear regression model $X_{j}|X_{-j}∼N(\alpha_{j}+\beta_{j}^{\text{T}}X_{-j},\omega_{j})$ where $\beta_{j}^{\text{T}}=\varsigma_{j}^{\text{T}}{\tilde{\Sigma }}_{j}^{-1}$, $\alpha_{j}=\mu_{j}-\beta_{j}^{\text{T}}{\tilde{\mu }}_{j}$ and $\omega_{j}=\sigma_{j}-\varsigma_{j}^{\text{T}}{\tilde{\Sigma }}_{j}^{-1}\varsigma_{j}$[2] p. 157. Using our notation for chained equations imputation (see under the subsection “Chained equations imputation” of the Methods section) *ψ*_*j *_= (*α*_*j*_,*β*_*j*_,*ω*_*j*_) and $\tilde{\psi_{j}}=({\tilde{\mu }}_{j},{\tilde{\Sigma }}_{j})$.

The joint prior for *ψ*_*j *_and ${\tilde{\psi }}_{j}$ derived from *p* (*θ*) is $p(\psi_{j},{\tilde{\psi }}_{j})=|\Sigma {|}^{-\kappa }\times |{\tilde{\Sigma }}_{j}|$[2] p. 158-159. So, using a standard result from matrix algebra for the determinant of a partitioned matrix,

$$\begin{array}{ll} p(\psi_{j},{\tilde{\psi }}_{j}) & ={\left(\sigma_{j}-\varsigma_{j}^{\text{T}}{\tilde{\Sigma }}_{j}^{-1}\varsigma_{j}\right)}^{-\kappa }\times |{\tilde{\Sigma }}_{j}{|}^{-\kappa }\times |{\tilde{\Sigma }}_{j}|\\ & =\omega_{j}^{-\kappa }\times |{\tilde{\Sigma }}_{j}{|}^{-(\kappa -1)}\\ & =p(\psi_{j})\times p({\tilde{\psi }}_{\left(j\right)}). \end{array}$$

Therefore, the non-informative margins condition of Proposition 1 is satisfied.

#### ***Example 2: Saturated multinomial distribution***

We now show for joint modelling imputation under a saturated multinomial model and a Dirichlet prior for *θ*, that the corresponding chained equations algorithm satisfies the non-informative margins condition of Proposition 1.

Consider *K* categorical variables *X* = (*X*_1_,…,*X*_*K*_), where each *X*_*j *_takes possible values 1,…,*I*_*j *_(*j* = 1,…,*K*). Variables *X* define a *K*-way contingency table. Let *c* = (*c*_1_,…,*c*_*K*_) denote a generic cell of the contingency table, *θ*_*c *_denote the cell probability *pr *(*X* = *c*) and *∂* denote the set of all cells of the contingency table. The joint distribution of *X* is a multinomial distribution with parameter *θ* = (*θ*_*c*_ : *c* ∈ *∂*) and index equal to 1.

Summing the table counts over variable *X*_*j *_produces a collapsed contingency table defined by variables *X*_-*j*_. Let *c*_-*j *_= (*c*_1_,…,*c*_*j*-1_,*c*_*j*+1_,…,*c*_*K*_) denote a generic cell of the collapsed table, ${\tilde{\psi }}_{c_{-j}}$ denote the cell probability *pr *(*X*_-*j *_= *c*_-*j*_) and ${\tilde{\partial }}_{j}$ denote the set of all cells of the collapsed table. The marginal distribution of *X*_-*j *_ is multinomial with parameter ${\tilde{\psi }}_{j}=\{{\tilde{\psi }}_{c_{-j}}:c_{-j}\in {\tilde{\partial }}_{j}\}$, where ${\tilde{\psi }}_{c_{-j}}=\overset{I_{j}}{\underset{c_{j}=1}{\sum }}\theta_{c}$. The conditional distribution of *X*_*j *_given *X*_-*j *_= *c*_-*j*_ is the multinomial distribution with parameters $\psi_{c_{-j}}=\{\text{pr}(X_{j}=c_{j}|X_{-j}=c_{-j}):c_{j}=1,\ldots ,I_{j}\}$. So, the full set of parameters for the conditional distribution of *X*_*j *_given *X*_-*j*_ is $\psi_{j}=\{\psi_{c_{-j}}:c_{-j}\in {\tilde{\partial }}_{j}\}$.

If the prior distribution for *θ* is Dirichlet with hyperparameter *α* = {*α*_*c*_ : *c* ∈ *∂*}, then the implied prior distributions for ${\tilde{\psi }}_{j}$ and *ψ*_*j *_are independent: the prior for ${\tilde{\psi }}_{j}$ is Dirichlet with hyperparameter ${\tilde{\alpha }}_{j}=\{{\tilde{\alpha }}_{c_{-j}}:c_{-j}\in {\tilde{\partial }}_{j}\}$, where ${\tilde{\alpha }}_{c_{-j}}=\overset{I_{j}}{\underset{c_{j}=1}{\sum }}\alpha_{c}$, and the prior distribution for *ψ*_*j *_is the product of the set of independent Dirichlet distributions $\left\{\psi_{c_{-j}}∼D(\alpha_{c_{-j}}):c_{-j}\in {\tilde{\partial }}_{j}\right\}$, where $\alpha_{c_{-j}}=\{\alpha_{c}:c_{j}=1,\ldots ,I_{j}\}$ is a subset of *α*[2] p. 256. Since the prior for $\left(\psi_{j},{\tilde{\psi }}_{j}\right)$ can be factored into independent distributions for *ψ*_*j *_and ${\tilde{\psi }}_{j}$, the non-informative margins condition of Proposition 1 is satisfied.

#### ***Example 3: Unsaturated multinomial distribution***

When the joint model is an unsaturated multinomial model, we give an example where the conditional and marginal parameters *ψ*_*j *_and ${\tilde{\psi }}_{j}$ are not distinct (see comment 1 of Proposition 1). Consequently the non-informative margins condition of Proposition 1 is not satisfied.

Consider *K* categorical variables *X* = (*X*_1_,…,*X*_*K*_) as described in the saturated multinomial example. Assume that all cell probabilities are positive, *θ* (*c*) gt; 0 for all *c*, to ensure that every multinomial distribution considered can be written as a log linear model and that all possible conditional distributions exist [28] p. 202. Let the joint model be the hierarchical log linear model that contains all two-way factors between the *K* variables and no higher order factors. We shall refer to this as the all two-way factor hierarchical model. Under this model, for any *j* the conditional distribution of *X*_*j *_given *X*_-*j *_follows a multinomial logistic regression model (or a logistic regression model when *X*_*j *_is binary) where the regression model includes variables *X*_-*j *_as main effects only (i.e. no interaction terms).

In generating class notation [29] the all two-way factor hierarchical model is written as [ 1 2]…[ 1 *K*]… [ (*K* - 1) *K*]. Any hierarchical log linear model for *X* can be represented by an undirected graph in which the graph vertices are the variables *X*_1_,…,*X*_*K *_and two vertices *X*_*g *_and *X*_*h *_are connected by an edge if and only if the log linear model contains two-factor or higher order terms involving variables *X*_*g *_and *X*_*h*_. Any model containing all two-factor terms is therefore represented by the complete graph. Asmussen and Edwards [29] state that two vertices in a graph are adjacent if there is an edge between them. For any subset *a* of the vertices, Asmussen and Edwards define the boundary of *a* to be the set of vertices that are not in *a* but that are adjacent to one or more vertices in *a*. So, for the complete graph, the boundary of *X*_*j *_is *X*_-*j*_. Theorem 2.3 of [29] states that a hierarchical log linear model is collapsible onto *X*_-*j *_if and only if the boundary of *X*_*j *_is contained in a generator of the hierarchical log linear model. Further, Theorem 4.1 of [29] states that if a hierarchical log linear model is not collapsible onto *X*_-*j*_, then parameters *ψ*_*j *_and ${\tilde{\psi }}_{j}$ are not distinct. For any *j*, *X*_-*j*_, the boundary of *X*_*j *_in the complete graph, contains all of the remaining *K* - 1 variables. When *K* ≥ 4, *X*_-*j*_ is not contained in any of the generators, [ 1 2]…[ 1 *K*]… [ (*K* - 1) *K*], of the all two-way factor hierarchical model, and hence the log linear model is not collapsible onto *X*_-*j*_. Therefore, parameters *ψ*_*j *_and ${\tilde{\psi }}_{j}$ are not distinct, and so the non-informative margins condition of Proposition 1 is not satisfied when *K* ≥ 4.

#### ***Example 4: General location model***

We give an example of a chained equations algorithm derived from joint modelling under a general location model that does not satisfy the non-informative margins condition of Proposition 1. Our simulation study is based on this example.

Suppose that the data on each individual consist of one incomplete binary variable *Y* and *K* - 1 continuous variables *W *= (*W*_1_,…,*W*_*K* - 1_)^T^, where one or more of the continuous variables are also incomplete. Let the joint distribution of *X* = (*Y*,*W*)^T^ be the general location model *Y* ∼ *Bernoulli *(*γ*) and *W* ∣ *Y* ∼ *N* (*μ*_0_ + *μ*_1_*Y*,*Σ*), for unknown parameters *θ* = (*γ*,*μ*_0_,*μ*_1_,*Σ*) [30]. Let the joint prior for *θ* be *p *(*θ*) = *γ*^*τ*-1^ (1-*γ*)^*ν*-1^ ∣ *Σ* ∣ ^-*κ*^ with hyperparameters *τ*,*ν* gt; 0 and κ∈ℚ.

From the multivariate normal example above it is straightforward to show that the non-informative margins condition of Proposition 1 holds for imputing any *W*_*j*_.

The conditional distribution of *Y* given *W*, *p*(*Y* ∣ *W*, *ψ*_*Y*_), is the logistic regression model with covariates *W*[25]. The marginal distribution of *W*, $p(W|{\tilde{\psi }}_{Y})$, can be written as a mixture of normal distributions

$$\begin{array}{ll} p(W|{\tilde{\psi }}_{Y}) & =\gamma p(W|Y=1,\mu_{0},\mu_{1},\Sigma )\\ & \;+(1-\gamma )p(W|Y=0,\mu_{0},\Sigma ). \end{array}$$

This cannot be parameterized more parsimoniously than ${\tilde{\psi }}_{Y}=(\gamma ,\mu_{0},\mu_{1},\Sigma )=\theta$. As *ψ*_*Y*_ is a function of *θ*, it is determined by $\theta ={\tilde{\psi }}_{Y}$.

Consequently, given ${\tilde{\psi }}_{Y}$ the parameters of the logistic regression model *ψ*_*Y *_are fully determined, and so ${\tilde{\psi }}_{Y}$ and *ψ*_*Y*_ are not distinct. Therefore, as discussed in comment 1 above, the non-informative margins condition of Proposition 1 does not hold.

Using the same argument, the non-informative margins condition of Proposition 1 does not hold for a chained equations algorithm derived from joint modelling under the restricted general location model, with cell probabilities restricted by the all two-factor hierarchical model (discussed in the unsaturated multinomial example) and cell means restricted to be a linear function of the categorical variables.

### Simulation study results

This section reports the results of the simulation study, where the chained equations conditional models were compatible with the same joint model but the non-informative margins condition of Proposition 1 was not satisfied.

**Figure 1:**
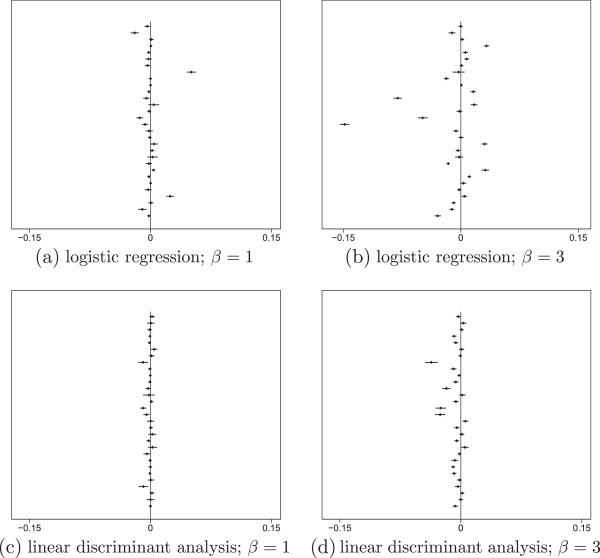
**Four forest plots of the posterior mean differences **${\bar{\beta }}^{b}-{\bar{\beta }}^{c}$**.** Each panel is a forest plot of ${\bar{\beta }}^{b}-{\bar{\beta }}^{c}$ for the first 30 datasets, 95% confidence intervals calculated using the Monte Carlo standard error. Panels **(a)** and **(b)**  correspond to binary variable *Y* imputed under the logistic regression model, with *β* = 1 and *β* = 3 respectively. Panels **(c)** and **(d)** correspond to *Y* imputed under the linear discriminant model, with *β* = 1 and *β* = 3 respectively.

Figures 1a and 1b show, for the first 30 of the 500 datasets, the value of ${\bar{\beta }}^{b}-{\bar{\beta }}^{c}$ (estimate of the order effect in one dataset) along with its 95% confidence interval, for the (standard) chained equations procedure (i.e., binary variable *Y* imputed under the logistic regression model). In a number of the datasets the 95% confidence intervals did not cross zero. Thus, there was clear evidence of order effects, with the magnitude of such effects varying between datasets. Such statistically significant evidence of an order effect occurred in 164 and 386 of the 500 datasets for *β* = 1 and *β* = 3 respectively. The range of absolute values of ${\bar{\beta }}^{b}-{\bar{\beta }}^{c}$ was larger for *β* = 3 than for *β* = 1. These results confirm that the chained equations procedure was not equivalent to any joint model procedure.

The average magnitudes of the order effects ${\bar{\beta }}^{b}$, ${\bar{\beta }}^{c}$ and $\left|{\bar{\beta }}^{b}-{\bar{\beta }}^{c}\right|$ over the 500 datasets are shown in Table 1. Consider the results for the chained equations algorithm (labelled LR). In keeping with Figure 1, the average magnitude of the order effect was larger for *β* = 3 than *β* = 1. The means of ${\bar{\beta }}^{b}$ and ${\bar{\beta }}^{c}$ did not differ systematically, consistent with the direction of the order effect being arbitrary and dataset dependent. Estimates ${\bar{\beta }}^{b}$ and ${\bar{\beta }}^{c}$ appeared to be equally good estimates of *β*.

**Table 1:** **Over 500 datasets, average of the complete case estimates, joint modelling imputation estimates and values of **${\bar{\beta }}^{b}$**, **${\bar{\beta }}^{c}$** and **$\left|{\bar{\beta }}^{b}-{\bar{\beta }}^{c}\right|$** for the chained equations algorithm and the modified chained equations algorithm, with confidence intervals [mean ± 1 *. *** **96 × (standard deviation ÷ *√ *** **500)]**

***β***	${\hat{\beta }}_{\text{CCA}}^{♭}$	${\hat{\beta }}_{\mathrm{JM}}^{♯}$		${\bar{\beta }}^{b}$	${\bar{\beta }}^{c}$	${|\bar{\beta }}^{b}-{\bar{\beta }}^{c}|$
1	1 · 01	0 · 97	*LR*^∇^	0 · 98	0 · 98	0 · 0040
	[0 · 93,1 · 09]	[0 · 89,1 · 05]		[0 · 90,1 · 06]	[0 · 90,1 · 06]	[0 · 0034,0 · 0046]
			*LDA*^*¶*^	0 · 97	0 · 97	0 · 0022
				[0 · 89,1 · 05]	[0 · 89,1 · 05]	[0 · 0020,0 · 0024]
3	3 · 00	2 · 90	LR	2 · 93	2 · 93	0 · 0166
	[2 · 91,3 · 09]	[2 · 82,2 · 98]		[2 · 85,3 · 02]	[2 · 85,3v· 02]	[0 · 0145,0 · 0188]
			LDA	2 · 89	2 · 89	0 · 0067
				[2 · 81,2 · 98]	[2 · 81,2 · 97]	[0 · 0062,0 · 0073]

*♭* complete case analysis estimate of *β* from the observed data only;

*♯* estimate of *β* from joint modelling imputation;

∇ logistic regression;

¶ linear discriminant analysis.

The forest plots of ${\bar{\beta }}^{b}-{\bar{\beta }}^{c}$ with 95% confidence intervals corresponding to the modified chained equations algorithm (i.e *Y* imputed under the linear discriminant model) are shown in Figures 1c and 1d. Order effects were smaller than in Figures 1a and b, but were still present because the modified algorithm did not use information about *W*_1_ and *W*_2_ when *Y* was missing. Out of the 500 datasets the number of datasets that showed statistically significant evidence of an order effect was 113 for *β* = 1 and 330 for *β* = 3.

From Table 1, the complete case estimates of *β* were unbiased. When *β* = 3 the linear discriminant analysis values ${\bar{\beta }}^{c}$ and ${\bar{\beta }}^{b}$, and the joint modelling imputation estimate ${\hat{\beta }}_{\text{JM}}$ were slightly biased towards the null. This bias was due to the prior (which was the same for imputation under the linear discriminant analysis model and joint modelling imputation under the general location model) and it disappeared when the sample size was increased to 1000 observations and when the simulation study was conducted using improper imputation; i.e using maximum likelihood estimates instead of Bayesian draws of parameters. For joint modelling imputation, and the standard and modified chained equations procedures changing their specifications (e.g., larger number of iterations, burn-in period) gave the same pattern of results as above (results not shown but available on request from the authors).

In preliminary simulation studies, when the non-informative margins condition was satisfied the results were consistent with a zero order effect (results not shown but available on request from the authors).

## Discussion

We have defined a non-informative margins condition which, together with compatibility of the conditional models, we have proved is sufficient for a chained equations algorithm to impute missing data from the predictive distribution of the missing data implied by the joint model and its prior distribution. Also, we have shown that compatibility of the conditional models is not alone a sufficient condition. In a scenario where the conditionals models were compatible but the non-informative margins condition failed, our simulation study showed that the distribution from which the final imputed values of the variables were drawn differed, in a dataset-dependent manner, according to the order in which the variables were updated in the chained equations sampler.

In work that is complementary to the finite-sample results presented in this paper, Liu et al. [19] identified sufficient conditions for chained equations imputation and imputation under a fully Bayesian model to be asymptotically equivalent; that is, for the supremum of the difference between the two imputation distributions to converge to zero as the sample size tends to infinity. This implies that when the non-informative margins condition is not satisfied but the conditional models are compatible with the same joint model, the order effects identified in our simulation study will disappear as the sample size tends to infinity.

In our simulation study the average magnitude of the order effects was small and did not induce bias. Given that chained equations imputation is a widely used approach to imputation, these results are somewhat reassuring. However, the scope of these simulations was limited and it remains possible that chained equations imputation could lead to more substantial bias in different situations; for example, when there are many partially observed variables.

When the non-informative margins condition does not hold we expect some loss of efficiency in general (because some information is discarded). However, in our simulation study we did not detect any sizable loss of efficiency. The issue of variance estimation for chained equations imputation is beyond the scope of this paper.

The advantage of chained equations imputation is that we do not need to specify the joint distribution of the variables. In cases where it is not known that there is a joint distribution, several methods for checking compatibility have been proposed (e.g., [16,31-35]). In practice, these methods are either limited to discrete distributions or are difficult to apply for multivariate distributions of more than 2 or 3 dimensions. This means that it may not be possible to check that the conditionals are compatible with the same joint model or that our non-informative margins condition holds true. van Buuren and other authors [3,18], in the examples they considered, concluded that chained equations is a robust approach even when the set of conditionals are not compatible with the same joint model. The findings of our simulation study support this body of work. Other studies [3,4,36,37] have compared chained equations and joint modelling, when missingness is multivariate, nonmonotone and ignorable, in settings which reflect real data (e.g. mixture of different types of variables, non-linear relationships and interactions between variables, semi-continuous variables). None of these studies has reported substantial differences in the performances of joint modelling imputation and chained equations imputation. Nonetheless many authors emphasize the need for further understanding of the theoretical underpinnings of the chained equations approach and the establishment of the robustness of the chained equations method (e.g. [3,7,14,38]).

## Conclusions

In finite samples, compatibility of the conditionals and our non-informative margins condition are sufficient for chained equations and joint modelling to yield imputations from the same predictive distribution. Furthermore, our simulation study demonstrated that, even in a simple setting, a chained equations procedure that does not satisfy the non-informative margins condition is not necessarily equivalent to a joint model procedure, even though when its conditional models are compatible.

When conditionals are incompatible or the non-informative margins condition is not satisfied, the distribution from which the imputed values are drawn can differ according to the order in which the variables are updated in the chained equations sampler, thereby introducing order effects.

Given the widespread use of chained equations imputation in medical research for datasets with different types of variables, researchers must be aware that order effects are likely to be ubiquitous. As noted by van Buuren [3], further work is needed to verify the robustness of chained equations to incompatibility of the conditional models in more general and realistic settings. Equally, future work could evaluate the robustness of chained equations imputation when the sample size is small-to-moderate, the conditionals are compatible and the non-informative margins condition is not satisfied.

## Competing interests

The authors declare that they have no competing interests.

## Authors’ contributions

IRW and JRC proposed the project. All authors contributed to the examples and/or the simulation study. RAH carried out the programming for the simulation study. RAH drafted the manuscript, and RAH, IRW and SRS edited the manuscript. All authors read and approved the final manuscript.

## Pre-publication history

The pre-publication history for this paper can be accessed here:

http://www.biomedcentral.com/1471-2288/14/28/prepub

## References

[B1] Rubin DB Multiple Imputation for Nonresponse in Surveys 1987 New York: John Wiley & Sons, Inc

[B2] Schafer JL Analysis of Incomplete Multivariate Data 1997 London: Chapman & Hall

[B3] van Buuren S **Multiple imputation of discrete and continuous data by fully conditional specification** Stat Methods Med Res 2007 16 219 242 10.1177/0962280206074463 17621469

[B4] Raghunathan TE Lepkowski JM van Hoewyk J Solenberger P **A multivariate technique for multiply imputing missing values using a sequence of regression models** Surv Methodol 2001 27 85 95

[B5] Gelman A Raghunathan TE **[Conditionally specified distributions: An introduction]: comment** Stat Sci 2001 16 268 269

[B6] van Buuren S Boshuizen HC Knook DL **Multiple imputation of missing blood pressure covariates in survival analysis** Statist Med 1999 18 681 694 10.1002/(SICI)1097-0258(19990330)18:6<681::AID-SIM71>3.0.CO;2-R 10204197

[B7] van Buuren S Groothuis-Oudshoorn K **mice: Multivariate Imputation by Chained Equations in R** J Stat Softw 2011 45 1 67

[B8] Morgenstern M Wiborg G Isensee B Hanewinkel R **School-based alcohol education: Results of a cluster-randomized controlled trial** Addiction 2009 104 402 412 10.1111/j.1360-0443.2008.02471.x 19207348

[B9] Mueller B Cummings P Rivara F Brooks M Terasaki R **Injuries of the head, face, and neck in relation to ski helmet use** Epidemiology 2008 19 270 276 10.1097/EDE.0b013e318163567c 18277163

[B10] Nash D Katyal M Brinkhof M Keiser O May M Hughes R Dabis F Wood R Sprinz E Schechter M Egger M **Long-term immunologic response to antiretroviral therapy in low-income countries: A collaborative analysis of prospective studies** AIDS 2008 22 2291 2302 10.1097/QAD.0b013e3283121ca9 18981768 PMC2794130

[B11] Souverein O Zwinderman A Tanck T **Multiple imputation of missing genotype data for unrelated individuals** Ann Hum Genet 2006 70 372 381 16674559 10.1111/j.1529-8817.2005.00236.x

[B12] Huo D Adebamowo C Ogundiran T Akang E Campbell O Adenipekun A Cummings S Fackenthal J Ademuyiwa F Ahsan H Olopade O **Parity and breastfeeding are protective against breast cancer in nigerian women** Br J Cancer 2008 98 992 996 10.1038/sj.bjc.6604275 18301401 PMC2266848

[B13] Wiles N Jones G Haase A Lawlor D Macfarlane G Lewis G **Physical activity and emotional problems amongst adolescents** Soc Psychiatry Psychiatric Epidemiol 2008 43 765 772 10.1007/s00127-008-0362-9 18438732

[B14] Azur M Stuart E Frangakis C Leaf P **Multiple imputation by chained equations: what is it and how does it work?** Int J Methods Psychiatric Res 2011 20 40 49 10.1002/mpr.329 PMC3074241 21499542

[B15] White IR Royston P Wood A **Multiple imputation using chained equations: Issues and guidance for practice** Stat Med 2011 30 377 399 10.1002/sim.4067 21225900

[B16] Arnold BC Press SJ **Compatible conditional distributions** J Am Statist Assoc 1989 84 152 156 10.1080/01621459.1989.10478750

[B17] Heckerman D Chickering DM Meek C Rounthwaite R Kadie C **Dependency networks for inference, collaborative filtering, and data visualization** J Mach Learn Res 2000 1 49 75

[B18] van Buuren S Brand JPL Groothuis-Oudshoorn CGM Rubin DB **Fully conditional specification in multivariate imputation** J Stat Comput Simulat 2006 76 1049 1064 10.1080/10629360600810434

[B19] Liu J Gelman A Hill J Su Y Kropko J **On the stationary distribution of iterative imputations** Biometrika 2013 doi: 10.1093/biomet/ast044

[B20] Little RJA Rubin DB Statistical Analysis with Missing Data 2002 New York: John Wiley & Sons, Inc

[B21] Tanner MA Wong WH **The calculation of posterior distributions by data augmentation** J Am Statist Assoc 1987 82 528 540 10.1080/01621459.1987.10478458

[B22] Arnold B Castillo E Sarabia J **Conditionally specified distributions an introduction** Stat Sci 2001 16 249 265 10.1214/ss/1009213728

[B23] Kirkwood B Sterne J Essential Medical Statistics 2003 Hoboken, New Jersey, US: Wiley-Blackwell

[B24] Albert J Bayesian computation with R 2009 Dordrecht, Heidelberg, London, New York: Springer

[B25] Efron B **The efficiency of logistic regression compared to normal discriminant analysis** J Am Statist Assoc 1975 70 892 898 10.1080/01621459.1975.10480319

[B26] Cox D Snell E Analysis of Binary Data. second edition 1989 London, UK: Chapman and Hall

[B27] van Buuren S Oudshoorn C Multivariate Imputation by Chained Equations (Mice V1.0 User’s Manual) 2000 http://www.stefvanbuuren.nl/publications/MICE\%20V1.0\%20Manual\%20TNO00038\%202000.pdf

[B28] Whittaker J Graphical models in applied multivariate statistics 1990 New York: John Wiley & Sons, Inc

[B29] Asmussen S Edwards D **Collapsibility and response variables in contingency tables** Biometrika 1983 70 567 578 10.1093/biomet/70.3.567

[B30] Olkin I Tate RF **Multivariate correlation models with mixed discrete and continuous variables** Ann Math Stat 1961 32 448 465 10.1214/aoms/1177705052

[B31] Arnold B Castillo E Sarabia J **Compatibility of partial or complete conditional probability specifications** J Stat Plann Inference 2004 123 133 159 10.1016/S0378-3758(03)00137-X

[B32] Ip E Wang Y **Canonical representation of conditionally specified multivariate discrete distributions** J Multivariate Anal 2009 100 1282 1290 10.1016/j.jmva.2008.11.010

[B33] Tian G Tan M Ng K Tang M **A unified method for checking compatibility and uniqueness for discrete conditional distributions** Commun Stat: Theory Methods 2009 38 115 129

[B34] Chen H **Compatibility of conditionally specified models** Stat Probability Lett 2010 80 670 677 10.1016/j.spl.2009.12.025 PMC2861368 20436935

[B35] Kuo K Wang Y **A simple algorithm for checking compatibility among discrete distributions** Comput Stat Data Anal 2011 55 2457 2462 10.1016/j.csda.2011.02.017

[B36] Horton N Kleinman K **Much ado about nothing: A comparison of missing data methods and software to fit incomplete data regression models** Am Stat 2007 61 79 90 10.1198/000313007X172556 17401454 PMC1839993

[B37] Yu LM Burton A Rivero-Arias O **Evaluation of software for multiple imputation of semi-continuous data** Stat Methods Med Res 2007 16 243 258 10.1177/0962280206074464 17621470

[B38] Kenward M Carpenter J **Multiple imputation: current perspectives** Stat Methods Med Res 2007 16 199 218 10.1177/0962280206075304 17621468

